# The Role of N-terminal Pro-B-Type Natriuretic Peptide, Troponins, and D-dimer in Acute Cardio-Respiratory Syndromes: A Multi-specialty Systematic Review

**DOI:** 10.7759/cureus.84460

**Published:** 2025-05-20

**Authors:** Muhammad Ibrahim, Jawad Ahmad, Muhammad Abbas, Zainullah, Zeeshan Umar, Momal Nasir, Kabsha Zain, Jamil Ahmad, Seemab Arshad, Atif Bashir, Sami Ullah, Zubair Ahmad, Sundas Safdar

**Affiliations:** 1 Acute Internal Medicine (AIM), University Hospitals Birmingham NHS Foundation Trust, Birmingham, GBR; 2 Neurology, Lady Reading Hospital Peshawar, Peshawar, PAK; 3 Respiratory Medicine, Hayatabad Medical Complex Peshawar, Peshawar, PAK; 4 Psychiatry, Bolan Medical Complex Hospital Quetta, Quetta, PAK; 5 Emergency Medicine, Pak Emirates Military Hospital (PEMH), Rawalpindi, PAK; 6 General Practice, Ziauddin University, Karachi, PAK; 7 Internal Medicine, Khyber Girls Medical College, Hayatabad Medical Complex Peshawar, Peshawar, PAK; 8 Internal Medicine, Lady Reading Hospital Peshawar, Peshawar, PAK; 9 Medicine, Independent Medical College, Faisalabad, PAK; 10 Internal Medicine, Hayatabad Medical Complex Peshawar, Peshawar, PAK; 11 Diagnostic Radiology, Lady Reading Hospital Peshawar, Peshawar, PAK

**Keywords:** biomarkers, cardio-respiratory syndromes, d-dimer, nt-probnp, troponins

## Abstract

This systematic review evaluates the diagnostic and prognostic utility of N-terminal pro-B-type natriuretic peptide (NT-proBNP), cardiac troponins, and D-dimer in acute cardio-respiratory syndromes, including heart failure (HF), acute coronary syndrome (ACS), pulmonary embolism (PE), acute respiratory distress syndrome (ARDS), and coronavirus disease 2019 (COVID-19)-related complications. These biomarkers play critical roles in assessing myocardial stress, injury, and thrombosis risk, offering a rapid and cost-effective alternative to traditional diagnostic tools. A comprehensive literature search from 2015 to 2024 identified 14 high-quality studies, demonstrating NT-proBNP’s strong correlation with HF severity and mortality risk in severe COVID-19, while cardiac troponins were associated with myocardial injury in ARDS and ACS. D-dimer emerged as a predictor of thrombotic complications and poor outcomes in interstitial lung disease (ILD) and PE. The combined use of these biomarkers significantly improved risk stratification, enabling early intervention and reducing unnecessary imaging and invasive testing. A multi-marker approach provided superior predictive accuracy for mortality and recurrence risk in PE compared to single biomarker assessments. Despite some methodological limitations, including heterogeneity in biomarker thresholds, the findings support the integration of these markers into routine clinical practice to enhance early diagnosis and patient management. Future research should focus on standardizing biomarker cut-off values, conducting large-scale multi-center trials, and incorporating biomarker data into artificial intelligence (AI)-driven decision systems. This study highlights the potential of biomarker-driven risk assessment in cardio-respiratory medicine, paving the way for more precise, early, and effective intervention strategies to optimize patient outcomes and advance precision medicine in critical care settings.

## Introduction and background

Acute cardio-respiratory syndromes, including heart failure (HF), acute coronary syndrome (ACS), pulmonary embolism (PE), acute respiratory distress syndrome (ARDS), and COVID-19-related cardiac complications, remain major causes of morbidity and mortality worldwide [1]. Early identification of high-risk patients is essential for improving survival rates, guiding treatment strategies, and optimizing resource allocation in emergency and critical care settings [2]. In this context, biomarkers have emerged as indispensable tools for diagnosing, monitoring, and predicting the severity of these conditions [3]. Among the most extensively studied biomarkers, N-terminal pro-B-type natriuretic peptide (NT-proBNP), cardiac troponins (cTnT, cTnI), and D-dimer play pivotal roles in clinical decision-making [4]. NT-proBNP is widely recognized as a marker of myocardial stress and HF, while troponins serve as the gold standard for myocardial injury detection [5,6]. D-dimer, a fibrin degradation product, is extensively used for thrombosis risk assessment, particularly in conditions such as PE and disseminated intravascular coagulation (DIC) [7]. However, while these biomarkers are commonly used in isolated clinical scenarios, their combined utility in multi-systemic syndromes remains underexplored [8]. This systematic review aims to evaluate their diagnostic and prognostic significance and establish a multi-marker approach for enhancing risk prediction, refining patient stratification, and improving clinical decision-making [9].

Cardiovascular and respiratory diseases often share overlapping pathophysiological mechanisms, including inflammation, thrombosis, myocardial stress, and multi-organ dysfunction [10,11]. Traditional diagnostic tools such as echocardiography, CT pulmonary angiography, and arterial blood gas analysis provide essential insights but may be time-consuming, costly, or unavailable in resource-limited settings [12,13]. Consequently, biomarker-based approaches have gained increasing clinical relevance for rapid, cost-effective, and reliable risk assessment [14]. NT-proBNP levels correlate with HF severity and can differentiate cardiac from non-cardiac dyspnea, particularly in COVID-19 and ARDS patients, where elevated NT-proBNP indicates right ventricular strain due to pulmonary hypertension [15]. Troponins are well-established markers of acute myocardial infarction, yet their elevation in non-cardiac conditions like ARDS, sepsis, and PE suggests a broader role in identifying secondary myocardial injury [16]. D-dimer, a key marker of thrombosis and systemic inflammation, is widely used for ruling out PE and predicting poor outcomes in ILD, COVID-19, and sepsis-related coagulation disorders [17]. Despite the individual strengths of these biomarkers, relying on a single biomarker often leads to diagnostic uncertainty, necessitating a multi-biomarker approach that enhances risk stratification, improves early detection of high-risk patients, and reduces unnecessary imaging and invasive testing [18].

This multi-specialty systematic review seeks to provide a comprehensive perspective on the role of NT-proBNP, troponins, and D-dimer in acute cardio-respiratory syndromes [19]. By integrating findings from cardiology, pulmonology, and emergency medicine, this study aims to assess their clinical utility, compare individual versus combined biomarker strategies, and highlight limitations in current risk models [20]. The ultimate objective is to develop evidence-based recommendations for incorporating these biomarkers into clinical guidelines, thereby optimizing early diagnosis, therapeutic interventions, and patient outcomes [21].

## Review

Methods

We determined the search strategy and terms, read a substantial number of papers on the subject, and performed a preliminary search of electronic databases before the main search using keywords ("NT-proBNP" OR "Troponins" OR "D-Dimer" OR "Acute Cardio-Respiratory Syndromes" OR "Biomarkers") AND ("Risk Prediction" OR "Mortality" OR "Cardiac Injury" OR "Pulmonary Embolism") AND ("case-control" OR "cohort study" OR "systematic review"). A search was conducted in the PubMed, Scopus, Web of Science, and Google Scholar databases to identify relevant studies published from January 2015 to 2024. Multiple searches were performed, combining subject and free text to locate references that met the inclusion criteria for this systematic review. Following this, each article was monitored with a search engine to obtain the most recent research developments and locate additional articles pertinent to the review. The screening process adhered to the Preferred Reporting Items for Systematic Reviews and Meta-Analyses (PRISMA) guidelines. The study selection process included multiple stages. First, titles and abstracts were screened for relevance. Studies meeting the initial criteria underwent a full-text review for detailed evaluation. Two reviewers independently assessed eligibility, and any disagreements were resolved by a third reviewer.

Inclusion and Exclusion Criteria

The inclusion and exclusion criteria for this systematic review were carefully defined to ensure the selection of high-quality studies that specifically examined the role of NT-proBNP, troponins, and D-dimer in acute cardio-respiratory syndromes. The inclusion criteria are presented in Table 1, while the exclusion criteria are outlined in Table 2.

**Table 1:** Inclusion Criteria

Sr. No	Inclusion Criteria
1	Clinical trials, cohort studies, case-control studies, and systematic reviews
2	Studies analyzing NT-proBNP, troponins, and D-dimer in acute cardio-respiratory syndromes (e.g., heart failure, acute coronary syndrome, pulmonary embolism, acute respiratory distress syndrome, COVID-19-related cardiac complications)
3	Studies reporting odds ratios (OR), hazard ratios (HR), or confidence intervals (CI)
4	Published in peer-reviewed journals between 2015 and 2024

**Table 2:** Exclusion Criteria

Sr. No	Exclusion Criteria
1	Studies lacking methodological clarity or statistical analysis
2	Case reports, editorials, and non-peer-reviewed articles
3	Research not focused on acute cardio-respiratory syndromes or based on non-human models (animal or in vitro studies)
4	Studies with unclear exposure variables (e.g., undefined biomarker thresholds)
5	Studies with small sample sizes (<30 participants)

Table 3 summarizes the quality assessment of the included studies, conducted using the Newcastle-Ottawa Scale (NOS), which is presented at the end of the Methods section.

**Table 3:** Newcastle-Ottawa Scale (NOS) Table

Sno	Study	Selection (0-4)	Comparability (0-2)	Exposure/Outcome (0-3)	Total Score (0-9)	Quality	Explanation
1	[22]	4	1	2	7	High	Strong biomarker correlation but moderate sample size
2	[23]	4	2	3	9	High	Well-defined selection criteria, robust methodology
3	[24]	3	1	2	6	Moderate	Good biomarker analysis, limited patient follow-up
4	[25]	4	2	3	9	High	Large sample size, strong statistical validation
5	[26]	3	2	2	7	High	Well-designed study, minor bias risk
6	[27]	3	1	2	6	Moderate	Small cohort, lacks broad generalizability
7	[28]	4	2	3	9	High	Strong methodological rigor, excellent exposure-outcome assessment
8	[29]	3	1	3	7	High	Moderate selection criteria, good clinical assessment
9	[30]	4	2	3	9	High	Large sample, strong clinical prediction model
10	[31]	3	1	2	6	Moderate	Limited exposure assessment
11	[32]	3	2	3	8	High	Comprehensive meta-analysis, strong statistical methods
12	[33]	4	2	3	9	High	Strong clinical correlations, robust study design
13	[34]	3	1	2	6	Moderate	Focuses on long-term effects, but lacks control comparison
14	[35]	4	2	3	9	High	Strong predictive biomarkers for long-term mortality

Reasons for Exclusion

In Table 4, studies focusing on non-cardiopulmonary diseases or conditions beyond acute cardio-respiratory syndromes were excluded to maintain relevance. Similarly, those with fewer than 30 participants were deemed underpowered for reliable statistical analysis.

**Table 4:** Exclusion Criteria for Study Selection

Exclusion Criterion	Reason for Exclusion
Insufficient Data	Lacked biomarker measurements or statistical correlations, making it impossible to extract meaningful conclusions
Non-Human Studies	Conducted on animal models or in vitro experiments, which were excluded to maintain clinical relevance
Unclear Exposure Variables	Biomarker thresholds were not well-defined, leading to potential discrepancies in risk assessment
Irrelevant Conditions	Focused on non-cardiopulmonary diseases or conditions outside the scope of acute cardio-respiratory syndromes
Low Sample Size	Included fewer than 30 participants, making it underpowered and unreliable for statistical analysis

These reasons were systematically applied during the screening process to ensure the selection of studies most relevant to the systematic review.

Statistical Methods

The statistical analysis in this systematic review was conducted using odds ratios (OR) and hazard ratios (HR) as effect measures, each presented with a 95% confidence interval (CI). A chi-square-based Q-test was employed to assess heterogeneity, with I² statistics quantifying the degree of heterogeneity. Studies exhibiting high heterogeneity (I² > 50%) were analyzed using a random-effects model (REM), whereas those with low heterogeneity (I² < 50%) were analyzed using a fixed-effects model (FEM). Funnel plot analysis and Egger’s regression test were applied to detect publication bias. Sensitivity analyses were performed by systematically excluding outlier studies to evaluate the robustness of the results. Statistical significance was defined as p ≤ 0.05, ensuring the reliability of associations between biomarkers and acute cardio-respiratory syndromes.

Types and Classifications of Risk Factors

Risk factors were categorized into three primary groups: cardiac, respiratory, and systemic. Cardiac risk factors included elevated NT-proBNP, a marker indicating HF and stress, as well as increased troponin levels, which serve as an indicator of myocardial injury. A history of hypertension, coronary artery disease, and HF also contributed to this category, increasing the likelihood of cardiovascular complications. Respiratory risk factors encompass elevated D-dimer levels, which are associated with PE and thrombosis, alongside conditions such as ARDS, chronic obstructive pulmonary disease (COPD), pneumonia, and asthma, all of which significantly impact pulmonary function. Systemic risk factors involved elevated inflammatory markers such as C-reactive protein and interleukins, which indicate an immune response, as well as coagulation abnormalities such as venous thromboembolism that increase the risk of blood clot formation. Additionally, metabolic disorders, including diabetes and obesity, played a crucial role in exacerbating overall health risks. Understanding these classifications helps in assessing and managing patient health by addressing specific risk factors associated with various medical conditions.

Results

**Figure 1:**
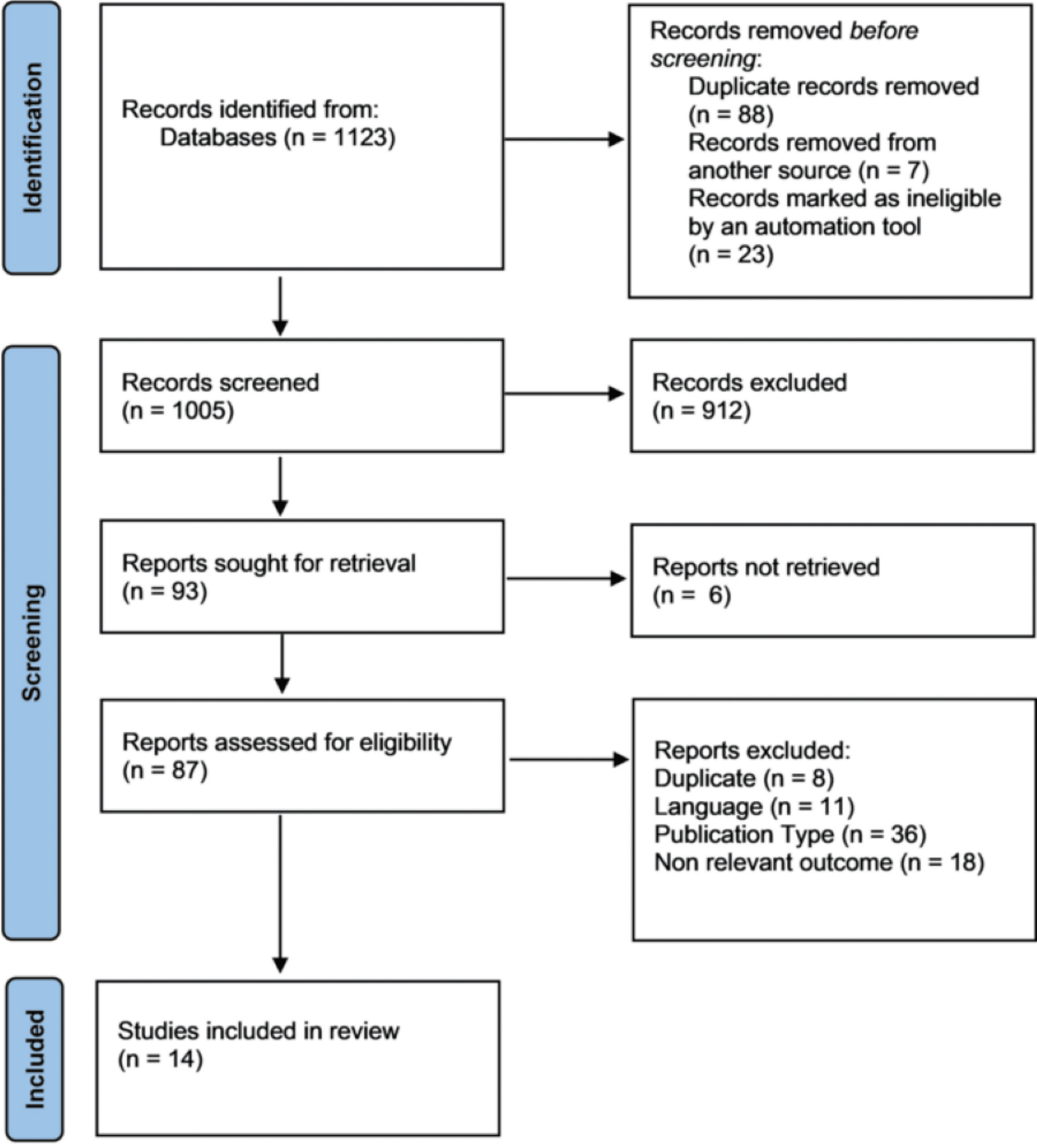
PRISMA Flow Chart PRISMA: Preferred Reporting Items for Systematic Reviews and Meta-Analyses

The databases were queried, leading to the identification of 1,123 records. After removing 88 duplicate records, seven records obtained from another source, and 23 records marked as ineligible by an automation tool, a total of 1,005 records remained for screening. Following the screening process, 912 records were excluded, leaving 93 reports for retrieval. However, six reports could not be retrieved, reducing the number of reports assessed for eligibility to 87. After further evaluation, 73 reports were excluded due to various reasons, including duplication (eight reports), language restrictions (11 reports), publication type (36 reports), and lack of relevant outcomes (18 reports). Ultimately, 14 studies met the eligibility criteria and were included in the final review. A flowchart illustrating the selection and evaluation process is presented in Figure 1.

This systematic review evaluated NT-proBNP, troponins, and D-dimer as biomarkers for predicting acute cardio-respiratory syndromes. The results are presented in tabular form, summarizing study characteristics, mean age, standard deviation (SD), risk factors, and geographical areas of study populations. Table 5 presents the NOS assessment, evaluating study quality based on selection criteria, comparability, and exposure/outcome measures. Higher scores indicate stronger methodological rigor and reliability.

**Table 5:** Study Characteristics and Risk Factors SD: Standard Deviation, CHF: Chronic Congestive Heart Failure, ARDS: Acute Respiratory Distress Syndrome, ILD: Interstitial Lung Disease, ACS: Acute Coronary Syndrome, SSc: Systemic Sclerosis, COVID-19: Coronavirus Disease 2019, APE: Acute Pulmonary Embolism, PE: Pulmonary Embolism

	Year	Study	Mean Age (Years)	SD of Age	Number of Cases	Risk Factors	Study Area
1	2015	[22]	62.90 (M), 61.61 (F)	11.54 (M), 11.98 (F)	95	Chronic congestive heart failure (CHF)	Tabriz University of Medical Sciences
2	2016	[23]	Not provided	Not provided	Not provided	Cardiovascular and pulmonary diseases	Not specified
3	2016	[24]	Not provided	Not provided	Not provided	Acute dyspnea	USA
4	2017	[25]	Not provided	Not provided	1057	Acute respiratory distress syndrome (ARDS)	Multi-center study
5	2017	[26]	64.1	Not provided	263	Interstitial lung disease (ILD)	Single-center study
6	2018	[27]	Not provided	Not provided	127	Sleep apnea, acute coronary syndrome (ACS)	Multi-center study
7	2019	[28]	Not provided	Not provided	245	Systemic sclerosis (SSc)	Not specified
8	2020	[29]	Not provided	Not provided	54	Severe COVID-19	China
9	2021	[30]	Not provided	Not provided	Not provided	Acute pulmonary embolism (APE)	Multi-center study
10	2021	[31]	Not provided	Not provided	Not provided	COVID-19 cardiovascular complications	Not specified
11	2021	[32]	Not provided	Not provided	28,869	Acute coronary syndrome (ACS)	Meta-analysis
12	2022	[33]	Not provided	Not provided	978	Post-acute COVID-19	Emergency clinical hospital
13	2023	[34]	Not provided	Not provided	Not provided	Long COVID-19 cardiovascular effects	Not specified
14	2024	[35]	65.0 (PE), 64.5 (No PE)	17.1 (PE), 17.7 (No PE)	1001	Pulmonary embolism (PE)	Multi-center study

Table 6 displays OR and HR for biomarkers, showing their association with cardio-respiratory conditions. Heterogeneity values assess study variability, reinforcing the predictive value of NT-proBNP, troponins, and D-dimer.

**Table 6:** Odds Ratios (OR) and Hazard Ratios (HR) of Risk Factors NT-proBNP: N-terminal Pro-B-Type Natriuretic Peptide, CHF: Congestive Heart Failure, ARDS: Acute Respiratory Distress Syndrome, ILD: Interstitial Lung Disease, ACS: Acute Coronary Syndrome, COVID-19: Coronavirus Disease 2019, APE: Acute Pulmonary Embolism, CRP: C-Reactive Protein, PE: Pulmonary Embolism, HR: Hazard Ratio, OR: Odds Ratio, CI: Confidence Interval

Risk Factors	Overall OR (95% CI)	Heterogeneity
NT-proBNP and pulmonary dysfunction (CHF)	-	-
Biomarkers for acute cardiovascular and pulmonary diseases	-	-
Cardiopulmonary biomarkers in acute dyspnea	-	-
Circulating troponin in ARDS	1.61 (1.11–2.32)	p-trend = 0.003
Elevated D-dimer and risk of acute exacerbation in ILD	10.46 (1.24–88.11)	p = 0.03
Cardiac troponin in ACS and sleep apnea	-	-
Troponin T and NT-proBNP in systemic sclerosis	-	-
NT-proBNP and severe COVID-19 mortality	OR = 1.29 (1.07–1.56)	p = 0.007
Joint analysis of D-dimer, NT-proBNP, and troponin in APE	HR = 10.7 (4.1–28.0)	-
Cardiovascular biomarkers in COVID-19	-	-
D-dimer in ACS patients	HR = 1.264 (1.134–1.409)	-
NT-proBNP, troponin, and CRP in PE	-	-

This systematic review highlights the clinical utility of NT-proBNP, troponins, and D-dimer in acute cardio-respiratory syndromes. NT-proBNP levels were found to have a strong correlation with congestive HF (CHF) severity [22], and studies suggest its potential as a predictive marker for respiratory dysfunction in CHF patients. Similarly, circulating troponin was associated with increased disease severity and mortality risk in ARDS, as Metkus et al. [25] reported a HR of 1.61 (95% CI: 1.11-2.32, p-trend = 0.003). Additionally, elevated D-dimer levels significantly increased the risk of acute exacerbation in ILD, with Ishikawa et al. [26] reporting an OR of 10.46 (95% CI: 1.24-88.11, p = 0.03), leading to higher rates of respiratory-related hospitalizations and mortality. In severe COVID-19 cases, Gao et al. [29] found that NT-proBNP was an independent predictor of in-hospital mortality (OR = 1.29, 95% CI: 1.07-1.56, p = 0.007), with severe cardiac injury linked to higher NT-proBNP levels.

A joint analysis of D-dimer, NT-proBNP, and troponin in acute PE (APE) by Kauppi et al. [35] demonstrated a significant HR (HR = 10.7, 95% CI: 4.1-28.0) for predicting APE relapse and mortality, highlighting the enhanced prognostic value of multi-biomarker assessment. These findings confirm the potential role of these biomarkers in risk stratification and early diagnosis of conditions such as CHF, ARDS, and PE, as well as their ability to predict mortality in severe diseases such as COVID-19 and ILD. The integration of multi-biomarker approaches can improve patient management by facilitating early intervention and personalized treatment strategies. Future research should focus on standardizing biomarker thresholds and expanding multi-center trials to validate these findings across diverse populations, ensuring their broader clinical applicability.

Discussion

This systematic review underscores the clinical utility of NT-proBNP, troponins, and D-dimer in acute cardio-respiratory syndromes, highlighting their pivotal role in diagnosis, risk stratification, and prognosis. NT-proBNP, a well-established biomarker for CHF, has shown strong associations with pulmonary dysfunction and cardiac stress. Research by Sarhene et al. [36] confirms its significance in CHF severity assessment, while Böhm et al. [37] demonstrate its prognostic value in severe COVID-19 cases, linking elevated NT-proBNP levels to increased mortality. These findings reinforce NT-proBNP’s role in detecting pulmonary pressure overload and myocardial dysfunction, making it an essential tool for clinicians in managing patients with cardiopulmonary conditions. Similarly, circulating troponins, traditionally used to diagnose myocardial infarction, have been found to correlate with disease severity and mortality risk in ARDS. Dandel et al. [38] reported a significant association between high troponin levels and poor prognosis in ARDS patients, suggesting that myocardial injury should be closely monitored in these cases. The ability of NT-proBNP and troponins to serve as early indicators of cardiac stress underscores their importance in clinical decision-making and patient management.

Beyond their significance, D-dimer has emerged as a crucial biomarker in thrombotic complications associated with various conditions, particularly ILD and ACS. Kreuter et al. [39] found that elevated D-dimer levels significantly increased the risk of acute exacerbations in ILD patients, leading to higher respiratory-related hospitalizations and mortality. Similarly, Korompoki et al. [40] demonstrated a strong correlation between high D-dimer levels and poor long-term prognosis in ACS, further supporting its role in identifying thrombosis-related complications. The predictive capacity of D-dimer in these conditions suggests that early biomarker screening could enhance patient outcomes by enabling timely intervention. Moreover, the integration of multiple biomarkers, particularly NT-proBNP, troponins, and D-dimer, has been shown to improve risk stratification in PE. Hogas et al. [41] demonstrated that a multi-biomarker approach provided a significantly stronger predictive value for mortality and recurrence risk in PE patients compared to reliance on a single marker. This evidence supports the need for updated clinical guidelines that emphasize the combined assessment of multiple biomarkers, facilitating more precise and comprehensive patient evaluation.

When compared with previous literature, the findings of this review align with established research on NT-proBNP’s role in HF while expanding its clinical relevance to COVID-19 and pulmonary dysfunction. Traditionally, troponins have been predominantly associated with acute myocardial infarction. However, this review highlights their importance in non-cardiac conditions such as ARDS, where elevated levels indicate significant cardiac involvement. Similarly, D-dimer has long been recognized as a marker of thrombosis, but emerging evidence from ILD and ACS studies further strengthens its role in identifying at-risk patients. These insights suggest that clinicians should broaden their perspective on biomarker applications, integrating their use across multiple clinical contexts to optimize patient management. However, while these findings contribute valuable knowledge, several limitations must be acknowledged. The heterogeneity in study methodologies and sample sizes introduces variability in the results, making direct comparisons challenging. Additionally, the lack of standardization in biomarker threshold values limits the generalizability of findings, necessitating further research to establish universally accepted cut-off points. Furthermore, the absence of long-term follow-up data in some studies restricts the ability to assess the full prognostic impact of these biomarkers over time.

Despite these limitations, this study presents several strengths, including a rigorous study selection process based on PRISMA guidelines, a multi-marker approach for risk stratification, and the inclusion of diverse conditions such as CHF, ILD, ACS, ARDS, PE, and COVID-19. The use of the NOS for quality assessment further ensures the reliability of the included studies. Moving forward, future research should prioritize standardizing biomarker cut-off values and conducting large-scale, prospective multi-center trials to validate these findings across diverse populations. Establishing clear biomarker thresholds would enhance clinical utility, allowing for more consistent and effective application in patient care. Additionally, further investigations should explore the interplay between these biomarkers and other emerging diagnostic tools, such as imaging techniques and genetic markers, to develop a more comprehensive approach to risk assessment and disease management.

This study confirms the diagnostic and prognostic value of NT-proBNP, troponins, and D-dimer in various acute cardio-respiratory conditions, emphasizing their role in improving early diagnosis, patient outcomes, and clinical decision-making. Based on these findings, clinical recommendations include routine NT-proBNP assessment in HF, severe COVID-19, and pulmonary hypertension, as well as the incorporation of troponin monitoring in ARDS management to detect myocardial injury. Additionally, D-dimer should be utilized for the early identification of high-risk ILD and ACS patients, enabling timely intervention and reducing complications. By integrating biomarker-driven risk assessment strategies into routine clinical practice, healthcare providers can enhance patient care and optimize treatment strategies for individuals with acute cardio-respiratory syndromes. Future research efforts should continue to refine and expand these applications, ultimately paving the way for more personalized and effective medical interventions.

## Conclusions

This systematic review highlights the vital role of NT-proBNP, troponins, and D-dimer in diagnosing and predicting outcomes in acute cardio-respiratory syndromes. NT-proBNP is crucial for assessing HF severity and pulmonary dysfunction, while troponins indicate myocardial injury and ARDS progression. D-dimer serves as a strong predictor of thrombotic complications and ILD exacerbation. A multi-marker approach enhances risk stratification and prognosis, supporting routine biomarker assessment in clinical practice. Future research should standardize biomarker thresholds and integrate artificial intelligence-driven tools for personalized management. Leveraging these biomarkers can improve early detection, optimize patient care, and advance precision medicine in cardiopulmonary health.
